# High- versus low-intensity internet interventions for alcohol use disorders (AUD): A two-year follow-up of a single-blind randomized controlled trial

**DOI:** 10.1016/j.invent.2023.100630

**Published:** 2023-05-24

**Authors:** Niels Eék, Christopher Sundström, Martin Kraepelien, Jesper Lundgren, Viktor Kaldo, Anne H. Berman

**Affiliations:** a Centre for Psychiatry Research, Department of Clinical Neuroscience, Karolinska Institutet, & Stockholm Health Care Services, Region Stockholm, Sweden; b University of Gothenburg, Department of Psychology, Sweden; c Linnaeus University Faculty of Health and Life Sciences, Sweden; d Uppsala University Department of Psychology, Sweden

**Keywords:** Alcohol, Alcohol use disorder, Cognitive behavior therapy, Long-term follow-up, Internet intervention

## Abstract

Alcohol Use Disorders (AUD) are widespread and have serious consequences, but are among the most undertreated mental disorders. Internet interventions have been found effective in treating AUD, but we know little about long-term outcomes, two years or more after treatment. This study explored 12- and 24-month outcomes in alcohol consumption following initial 6-month improvements after a therapist-guided high-intensity internet intervention and an unguided low-intensity internet intervention among individuals with alcohol use disorder. Between-group comparisons were analyzed, as well as within-group comparisons with (1) pre-treatment measurements (2) post-treatment measurements. Participants consisted of a general population sample of internet help-seekers in Sweden. A total of 143 adults (47% men) with a score of 14 (women)/16 (men) or more on the Alcohol Use Disorders Identification Test, alcohol consumption of 11 (women)/14 (men) or more standard drinks the preceding week and ≥ 2 DSM-5 alcohol use disorder (AUD) criteria based on a diagnostic interview were included. The high- and low-intensity internet interventions (*n* = 72 and *n* = 71 respectively) consisted of modules based on relapse prevention and cognitive-behavioral therapy. The primary outcome was self-reported alcohol consumption in the preceding week measured as (1) number of standard drinks and (2) number of heavy drinking days. Attrition from self-reported questionnaires was 36% at the 12-month follow-up and 53% at the 24-month follow-up. No significant between-group differences occurred in outcomes at either long-term follow-up. Regarding within-group differences, compared to pre-treatment, alcohol consumption was lower in both high- and low-intensity interventions at both long-term follow-ups [within-group standard drinks effect sizes varied between *g* = 0.38–1.04 and heavy drinking days effect sizes varied between *g* = 0.65–0.94]. Compared to post-treatment, within-group alcohol consumption in the high intensity intervention increased at both follow-ups; for the low-intensity intervention, within-group consumption decreased at 12-month follow-up, but did not differ compared to post-treatment at 24 months. Both high- and low-intensity internet interventions for AUD were thus associated with overall reductions in alcohol consumption at long term follow-ups, with no significant differences between the two. However, conclusions are hampered by differential and non-differential attrition.

## Highlights

• Internet interventions for alcohol problems may be an effective clinical tool.
• Evaluations of more than one year are rare.
• Long-term effects of guided and unguided internet interventions were investigated.
• No differences in drinking were found at one and two year follow-ups.
• There was major both differential and non-differential attrition.

## 1 Introduction

Alcohol Use Disorders (AUD) (Connor et al., 2016) are highly prevalent, with serious consequences for both individuals and society (Rehm et al., 2010), and they are also among the most undertreated mental disorders, with fewer than 15% seeking treatment within the health care system (Cohen et al., 2007; Degenhardt et al., 2017; Probst et al., 2015). Reported reasons for not seeking treatment are plentiful (Probst et al., 2015; Rogers et al., 2019; Wallhed Finn et al., 2014) and the most common explanation for not seeking treatment is that AUD is severely stigmatized, inciting more social rejection than other psychiatric conditions (Kilian et al., 2021). Calculations show that if the number of individuals with AUD who received treatment doubled in comparison to current levels, alcohol-related mortality would decrease by 13% for men and 9% for women (Rehm and Roerecke, 2013). Thus, if treatment access were to increase, not only would those with AUD experience a reduction of the most common negative effects (i.e., relationship and/or workplace problems, somatic illness) they would also reduce the risk of early death. It is therefore an urgent matter to increase the availability of treatment that suits the different needs of individuals with AUD.

One of the most well-established and researched psychological treatments for AUD is relapse prevention, which is a form of cognitive behavior therapy (CBT) (Witkiewitz and Marlatt, 2004). Although relapse prevention is a standard component of treatment offered at addiction clinics, few persons with AUD actually access relapse prevention interventions due to the low level of treatment seeking in this population (e.g., Probst et al., 2015). Internet interventions are increasingly seen as an alternative to traditional face-to-face treatment, since they are highly accessible, eliminate geographical barriers and can attract people who otherwise would not seek help because of the stigma related to physically visiting a clinic (Kilian et al., 2021). Additional advantages are that they track participants' state of health with regular symptom questionnaires that also monitor treatment progress and risk of self-harm (Andersson et al., 2019b).

Internet interventions fall into two major categories. The first category is public health-oriented low-intensity internet interventions (LIIIs) aimed at the general population, often automatized and delivered without any human guidance (Grist and Cavanagh, 2013), most often based on the concept of “brief interventions” (Kaner et al., 2017; Riper et al., 2018). The number of digital brief interventions is rapidly increasing (Schaub et al., 2020), and their main advantages from a public health perspective is the low cost due to the possibility of administering the intervention to a virtually unlimited number of people. Historically, LIIIs for alcohol problems have been *aimed* at those with hazardous rather than severe drinking levels, even though LIIIs in practice often seem to *reach* those with severe drinking problems (Berman et al., 2020; Riper et al., 2018; Sinadinovic et al., 2014). The second major category of internet interventions consists of guided high-intensity internet interventions (HIIIs), with the content usually based on traditional CBT, and typically delivered with human guidance via asynchronous text messages (Andersson et al., 2019a). The main argument for the HIII concept has been to offer low-threshold access to treatment that matches even more severe diagnosed conditions (e.g., for depression), and to increase patients' access to manual-based therapy while minimizing therapist time (Andersson, 2009; Andersson et al., 2019a). HIIIs have mostly been studied for depression and anxiety, with meta-analysis indicating no difference in outcomes between internet interventions and face-to-face therapy (Andersson et al., 2019b). Internet interventions for alcohol problems, both LIIIs and HIIIs, have shown to be effective (Riper et al., 2018). As most studies on internet interventions for alcohol problems have been LIII, we decided to investigate whether a guided HIII could be more effective than an unguided LIII in the treatment of AUD in a randomized controlled trial. We noted sharp alcohol reductions in both groups at the 6-month follow-up, but identified no significant differences between HIII and LIII (Sundström et al., 2020).

Little data is available regarding long-term follow-up of internet interventions for AUD. A meta-analysis from 2018 (Riper et al., 2018) examined 19 different internet interventions for adult problem drinking, most with short-term follow-up of 6 months. Some studies have been identified investigating long-term effects of internet interventions for AUD, but follow up has been limited to a maximum of 12 months, and showed no differences in drinking outcomes between different interventions (e.g., Bischof et al., 2008; Sinadinovic et al., 2014). To our knowledge no findings have been published concerning follow-ups of two years or more in studies based solely on internet interventions for AUD.

### 1.1 Aims

In this long-term follow up of a randomized controlled study on two CBT-based internet interventions for AUD (Sundström et al., 2020), we conduct an investigation of alcohol consumption outcomes after 12 and 24 months. We note the previously shown lack of differences in between-group outcomes at 6-month follow-up, as well as the prior significant reductions in alcohol consumption within both groups at 6 months. However, in view of the dearth of research reporting long-term follow-ups in such trials, we maintain an exploratory stance and abstain from formulating directional hypotheses regarding maintenance, decrease or increase in consumption after 12 and 24 months, in either or both groups.

The specific aims of the study are threefold. The first aim is to explore any between-group differences, comparing HIII to LIII, at 12- and 24-month follow-ups. The second aim is to investigate within-group alcohol consumption compared to pre-treatment measurements. The third aim is to investigate within-group alcohol consumption compared to post-treatment measurements.

## 2 Methods

### 2.1 Trial design

This was a long-term follow-up of a previously reported randomized controlled trial of two different methods of internet interventions based on relapse prevention for alcohol use disorder, where participants (*N* = 143) were allocated to High Intensity Internet Intervention (HIII, *n* = 72) or a Low Intensity Internet Intervention (LIII, *n* = 71), followed up at post-treatment and 6 months post-randomization (Sundström et al., 2020). The previously published short-term evaluation also included a waiting-list with 23 participants, who were offered treatment after their 13-week waiting period, with no long-term follow-up data collection. Recruitment occurred between January 2016 and February 2017. Participants randomized to either of the interventions were blinded. This long-term explorative follow-up evaluated outcomes at 12- and 24-months post-randomization; i.e., 6 and 18 months after the 6-month follow-up reported earlier. Participants provided informed consent during initial recruitment, and follow-up data for the analyses reported herein were collected between May 1, 2017 and August 11, 2019.

### 2.2 Recruitment and participants

**Fig. 1:**
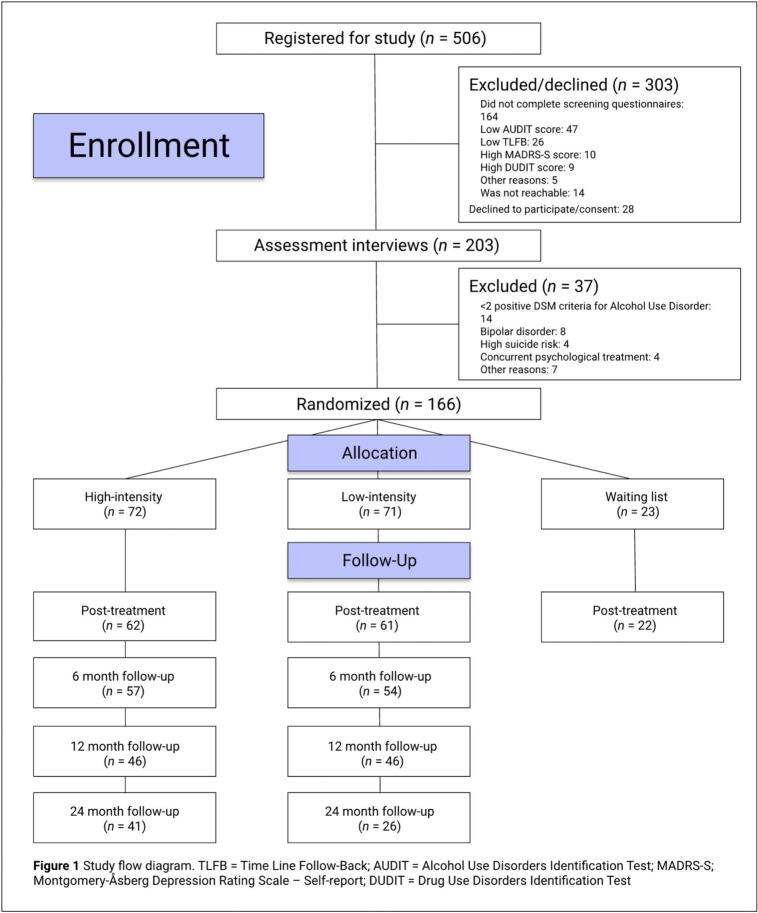
Study flow diagram. TLFB = Time Line Follow-Back; AUDIT = Alcohol Use Disorders Identification Test; MADRS-S; Montgomery-Åsberg Depression Rating Scale – Self-report; DUDIT = Drug Use Disorders Identification Test.

Participants were internet help-seekers, recruited via online self-referral that began with online screening and was followed by a structured diagnostic psychiatric telephone interview for those potentially eligible; see Fig. 1 for the study flowchart. Online ads were used for recruitment, including Google Adwords, Facebook and the Remente smartphone app. Inclusion criteria were: age ≥ 18 years, scoring ≥14 (females)/ ≥ 16 (males) on the Alcohol Use Disorders Identification Test (AUDIT) (Saunders et al., 1993), consuming ≥11 (females)/≥ 14 (males) standard drinks of alcohol in the preceding week and being diagnosed with an AUD, defined as two or more positive DSM-5 criteria for AUD. To establish an AUD diagnosis, telephone interviews were performed using the Structured Clinical Interview for DSM-IV axis I disorders (SCID-I) (Kranzler et al., 1996), adapted to DSM-5 criteria. Exclusion criteria were severe depression (MADRS ≥ 30p) (Svanborg and Åsberg, 2001), acute suicidal ideation, illicit drug use problems (DUDIT ≥ 8p) (Berman et al., 2017) insufficient skills in Swedish language, reading/writing difficulties, concurrent psychological treatment, and severe psychiatric comorbidity, defined as psychosis, bipolar disorder in active relapse and PTSD. Comorbid psychiatric disorders were assessed with the Mini International Neuropsychiatric Interview (MINI) (Sheehan et al., 1998).

### 2.3 Interventions

Both interventions (LIII and HIII) were twelve weeks long and based on the CBT model relapse prevention, but with some differences, detailed below. The platform was hosted on a server with encrypted traffic and authentication login function to guarantee participant confidentiality.

#### 2.3.1 High-intensity internet intervention

In the HIII, the ePlus program was used, which has been previously evaluated twice (Sundström et al., 2017, Sundström et al., 2020). In ePlus, therapists are urged to deliver approximately 15 min of guidance per week via written asynchronous messages. The ePlus program consists of 13 modules with three to four pages of text and short videos, and the participants completed homework assignments in each module. All participants had contact with a therapist using a secure built-in message system. For more detailed information about the content and method, see the earlier publication (Sundström et al., 2020).

#### 2.3.2 Low-intensity internet intervention

In the LIII, the eChange program was used, different versions of which have been evaluated in several trials (Blankers et al., 2011; Johansson et al., 2017; Sundström et al., 2016, Sundström et al., 2020). The program consists of eight modules with one to two pages of text per module. Participants were instructed to complete homework assignments and were consecutively granted access to new modules each week. There was no therapist guidance or other contact with the research team. During weeks 8–11 participants received no new modules, but weekly text messages reminded participants to log in.

### 2.4 Measures

All participants completed self-rated measurements online on eight occasions: at screening, just before treatment start, at weeks 4 and 8 during treatment, directly after treatment and again at 6-, 12- and 24-month follow-ups post-randomization. Only the primary outcome (see below) was measured pre-treatment and at weeks 4 and 8, while all outcome measures were completed at screening, post-treatment and 6-, 12- and 24-month follow-ups. As a final question in the 12- and 24-month follow-up outcome measurement, participants were asked if they would also agree to participate in a telephone interview, which included the same diagnostic questions from MINI and SCID-I as in the screening assessment.

The primary outcome was alcohol consumption, specifically (1) total number of standard drinks (12 g of ethanol) the preceding 7 days and (2) number of heavy drinking days (HDD), defined as ≥5 (men) or ≥4 (women) standard drinks on a single day, during the preceding 7 days. Alcohol consumption was measured using a self-report version, online version of the Timeline Follow-Back (Sobell et al., 1979), given studies that show good psychometric properties for online Timeline Follow-Back measures (Hoeppner et al., 2010; Pedersen et al., 2012; Rueger et al., 2012).

Secondary outcome measures included the Alcohol Use Disorders Identification Test (AUDIT) (Saunders et al., 1993) to assess changes in alcohol problems, the Alcohol Abstinence Self Efficacy Scale (AASES) (Berman et al., 2010) to assess changes in self-efficacy and the Penn Alcohol Craving Scale (PACS) (Flannery et al., 1999), all three alcohol-related questionnaires. The Montgomery-Åsberg Depression Rating Scale self-report (MADRS-S) (Svanborg and Åsberg, 2001) was used to assess changes in depression and the Generalized Anxiety Disorder Scale (GAD-7) was used to assess changes in anxiety (Spitzer et al., 2006). The Euroqol-5D (EQ-5D) (Herdman et al., 2011) was used to assess changes in quality of life, but has not been analyzed since one part of the measure was lacking due to technical reasons. The SCID-I and MINI were used in the diagnostic interviews to assess AUD diagnosis and changes in AUD criteria count and severity over time, as recommended in previous research (Kiluk et al., 2018).

### 2.5 Statistical analyses

We conducted all analyses in SPSS version 27 (IBM Corp., Armonk, NY, USA). Generalized estimating equations (GEE) with an unstructured working correlation matrix, using a negative binomial model with log-link (Horton et al., 2007) and a 2 (intervention) × 8 (screening, pre-treatment, two measurements mid treatment (“mid1” and “mid2”), post treatment, 6-, 12- and 24-month follow ups, were used to assess interaction effects on primary outcomes. The same GEE analysis was used to calculate within-group effects on primary outcomes, comparing pre- and post-treatment levels to long-term follow-ups. The GEE was also used to evaluate effects on secondary outcomes, but with a normal model instead of the negative binomial model, since the secondary outcomes are regarded as normally distributed, contrary to alcohol consumption.

In a sensitivity analysis, the AASE and the total number of anxiety syndromes at screening were included as covariates, as these variables correlated highly with both alcohol consumption change over time and “missingness”, i.e., the tendency for data to be missing, at the long-term follow-ups. This increased the GEE-model's ability to perform estimations despite missing data.

A dropout analysis was conducted by comparing number of standard drinks and AUDIT scores at screening between active and non-active participants at the 12- and 24-month follow-ups.

## 3 Results

The 143 participants had a mean age of 52.8 years (SD = 11.1), and 47% were male. There were no significant differences between groups on baseline characteristics. For a table showing baseline characteristics, see the previously published short-term evaluation (Sundström et al., 2020).

Attrition was defined as not participating in the self-report follow-up questionnaires. Attrition rates at 12- and 24-month follow-ups for HIII and LIII were 36 and 53%, respectively, with no significant difference between the two intervention groups at 12-month follow-up (χ^2^ = 0.13, *p* = .911), but with a significant difference between the two groups at 24-month follow-up, where HIII had a 43% attrition rate, and LIII had a 63% attrition rate (χ^2^ = 5.93, *p* = .015). Four participants (one in HIII and three in LIII) did not complete pre-treatment measurements and made no subsequent attempts to access the treatment. In the dropout analyses, number of standard drinks was not significantly associated with dropout, either at the 12-month (*p* = .320) or at the 24-month follow-up (*p* = .460). AUDIT scores were not significantly associated with dropout at the 12-month follow-up (*p* = .149), but the association was significant at the 24-month follow-up (*p* = .012).

### 3.1 Primary outcomes

**Fig. 2:**
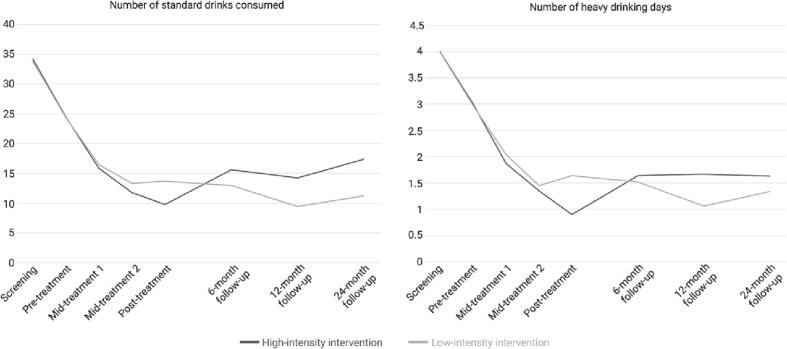
Number of estimated standard drinks consumed (left panel) and number of estimated heavy drinking days (right panel) during preceding calendar week before, during, after treatment and at short- and long-term follow-ups.

See Fig. 2 for a visualization of changes in alcohol consumption at all assessment points for both groups, expressed in weekly consumption of standard drinks and number of heavy drinking days.

#### 3.1.1 Were there any differences between HIII and LIII at long-term follow-up?

Comparisons between HIII and LIII showed that no significant differences occurred between the two groups at the 12- and 24-month follow-ups in either of the primary outcome measurements, see Table 1.

**Table 1:** GEE analysis showing estimated means of the primary outcomes standard drinks of alcohol consumed and heavy drinking days the previous week. Differences and effect sizes within groups, with no control group, between pre-treatment and 12- and 24-month follow-up, respectively.

Primary outcomes		Pre	12-Month follow-up	Sign	Effect size	24-Month follow-up	sign	Effect size
Drinks per week	HIII, m (SE)	24.51 (2.33)	14.24 (2.01)	*p* ≤0.001	*g* = 0.59	17.37 (2.77)	*p* = .021	*g* = 0.38
	LIII, m (SE)	24.39 (1.99)	9.47 (1.57)	*p* ≤0.001	*g* = 1.04	11.23 (1.87)	*p* ≤0.001	*g* = 0.89
Heavy drinking days	HIII, m (SE)	3.01 (0.26)	1.67 (0.27)	*p* ≤0.001	*g* = 0.65	1.63 (0.32)	*p* ≤0.001	*g* = 0.65
	LIII, m (SE)	2.98 (0.29)	1.02 (0.21)	*p* ≤0.001	*g* = 0.94	1.34 (0.29)	*p* ≤0.001	*g* = 0.75

#### 3.1.2 What was the level of alcohol consumption at long-term follow-up compared to pre-treatment measurements?

Within-group differences over time from pre-treatment measurement to long-term follow-ups, showed that both groups had significant alcohol reductions in drinks per week and heavy drinking days, at both 12- and 24-month follow-ups, see Table 2.

**Table 2:** GEE analysis showing estimated means of the primary outcomes standard drinks of alcohol consumed and heavy drinking days the previous week. Differences and effect sizes within groups, with no control group, between post-treatment and 12- and 24-month follow-up, respectively.

Primary outcomes		Post	12-Month follow-up	Sign	Effect size	24-Month follow-up	Sign	Effect size
Drinks per week	HIII, m (SE)	9.77 (1.37)	14.24 (2.01)	*p* = .026	*g* = −0.37	17.37 (2.77)	*p* = .001	*g* = −0.52
	LIII, m (SE)	13.69 (1.84)	9.47 (1.57)	*p* = .018	*g* = 0.33	11.23 (1.87)	*p* = .178	*g* = 0.19
Heavy drinking days	HIII, m (SE)	0.9 (0.17)	1. 67 (0.27)	*p* = .002	*g* = −0.49	1.63 (0.32)	*p* = .021	*g* = −0.46
	LIII, m (SE)	1.64 (0.24)	1.06 (0.20)	*p* = .014	*g* = 0.35	1.34 (0.29)	*p* = .284	*g* = 0.17

HIII = High-Intensity Internet Intervention; LIII = Low-Intensity Internet Intervention; SE = standard error.

#### 3.1.3 What was the level of alcohol consumption at long-term follow-up compared to post-treatment measurements?

Within-group differences over time from post-treatment measurement to long-term follow-ups, showed that participants in HIII had significantly increased alcohol consumption as compared to post, both in drinks per week and heavy drinking days, at 12- and 24-month follow-ups. Participants in LIII on the other hand, had significantly decreased alcohol consumption both in drinks per week and heavy drinking days at 12-month follow-up, but no change was evident at the 24-month follow-up, see Table 3.

**Table 3:** GEE analysis showing pairwise comparisons of estimated means of the primary outcomes standard drinks of alcohol consumed and heavy drinking days the previous week between HIII and LIII across all time points.

Primary outcomes		Screening	Pre	Mid1	Mid2	Post	6-Month	12-Month	24-Month
Drinks per week	HIII, m (SE)	34.24 (2.02)	24.51 (2.33)	15.86 (2.10)	11.78 (1.50)	9.77 (1.37)	15.62 (1.86)	14.24 (2.01)	17.37 (2.77)
	LIII, m (SE)	33.85 (1.94)	24.39 (1.99)	16.48 (1.65)	13.31 (1.84)	13.69 (1.84)	12.99 (1.90)	9.47 (1.57)	11.23 (1.87)
	Sign.	*p* = .889	*p* = .970	*p* = .815	*p* = .521	*p* = .087	*p* = .323	*p* = .062	*p* = .066
	Betwen-group effect size	*g =* −0.02	*g =* −0.01	*g =* 0.04	*g =* 0.14	*g =* 0.31	*g =* −0.19	*g =* −0.39	*g =* −0.41
Heavy drinking days	HIII, m (SE)	4.00 (0.23)	3.01 (0.26)	1.87 (0.24)	1.35 (0.22)	0.90 (0.17)	1.64 (0.26)	1.67 (0.27)	1.63 (0.32)
	LIII, m (SE)	3.99 (0.25)	2.98 (0.29)	2.04 (0.24)	1.45 (0.30)	1.64 (0.24)	1.52 (0.27)	1.06 (0.20)	1.34 (0.29)
	Sign.	*p* = .967	*p* = .941	*p* = .631	*p* = .804	*p* = .013	*p* = .739	*p* = .072	*p* = .507
	Effect size	*g =* −0.01	*g =* −0.01	*g =* 0.09	*g =* 0.06	*g =* 0.46	*g =* −0.06	*g =* −0.38	*g =* −0.16

HIII = High-Intensity Internet Intervention; LIII = Low-Intensity Internet Intervention; SE = standard error.

### 3.2 Secondary outcomes

#### 3.2.1 Change in AUD criteria count and severity over time

At baseline screening, the mean total DSM-5 AUD criteria count was 6.6 (SD = 2.0), see Table 4, with no significant difference between HIII and LIII.

**Table 4:** Number of AUD criteria and comparison of psychiatric diagnosis between high- with low intensity interventions at baseline and long-term follow-ups, obtained from diagnostic telephone interviews.

		Screening	12-Month	24-Month
Number of AUD criteria	HIII, m (SD)	6.6 (1.83)	4.7 (3.13)	3.4 (3.19)
	LIII, m (SD)	6.6 (2.10)	3.8 (2.74)	3.1 (2.90)


Psychiatric comorbidity (MINI)		Screening	12-Month	24-Month
Any psychiatric comorbidity	HIII, n (%)	28 (38.9)	6 (26.1)	6 (31.6)
	LIII, n (%)	30 (42.9)	4 (19.0)	4 (26.7)
	χ^2^	0.231	0.310	0.097
	Sign.	*p* = .631	*p* = .578	*p* = .755
Depression	HIII, n (%)	19 (26.4)	1 (4.3)	3 (15.8)
	LIII, n (%)	14 (20.0)	0 (0.0)	3 (20.0)
	χ^2^	0.812	0.934	0.102
	Sign.	*p* = .367	*p* = .334	*p* = .749
Agoraphobia	HIII, n (%)	9 (12.5)	2 (8.7)	3 (15.8)
	LIII, n (%)	10 (14.3)	2 (9.5)	2 (13.3)
	χ^2^	0.98	0.009	0.040
	Sign.	*p* = .755	*p* = .924	*p* = .841
Generalized anxiety disorder	HIII, n (%)	6 (8.3)	2 (8.7)	1 (5.3)
	LIII, n (%)	7 (10.0)	0 (0.0)	2 (13.3)
	χ^2^	0.119	1.913	0.679
	Sign.	*p* = .731	*p* = .167	*p* = .410
Social phobia	HIII, n (%)	5 (6.9)	1 (4.3)	0 (0.0)
	LIII, n (%)	6 (8.6)	0 (0.0)	0 (0.0)
	χ^2^	0.131	0.934	–
	Sign.	*p* = .717	*p* = .334	*p* = –
Other	HIII, n (%)	3 (4.2)	2 (8.7)	3 (15.8)
	LIII, n (%)	10 (14.3)	2 (9.5)	4 (26.7)
	χ^2^	4.370	0.009	0.607
	Sign.	*p* = .037	*p* = .924	*p* = .436

HIII = High-Intensity Internet Intervention; LIII = Low-Intensity Internet Intervention; other psychiatric comorbidity = bipolar disorder, panic disorder, obsessive compulsive disorder, post-traumatic stress disorder, psychosis, anorexia nervosa, ulimia nervosa, anti-social personality disorder; SE = standard error.

**Fig. 3:**
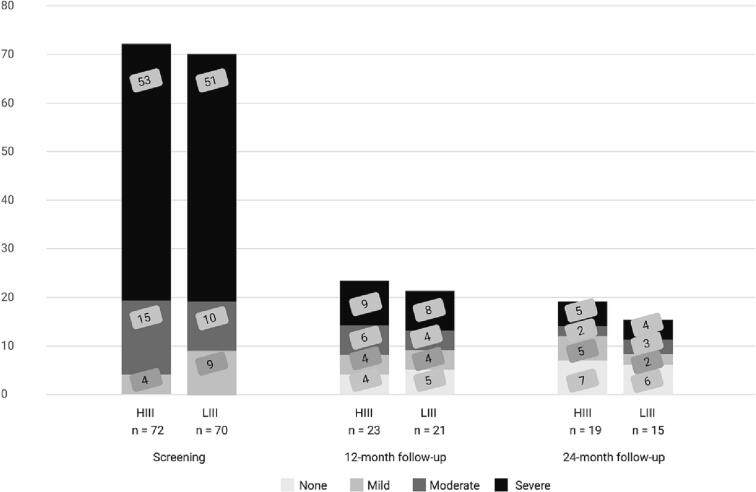
Percentage of participants in each AUD severity category at each time point. Mild (2–3 criteria), moderate (4–5 criteria), or severe (6 or more criteria).

AUD is classified into three severity categories: mild (2–3 criteria), moderate (4–5 criteria), or severe (6 or more criteria, maximum 11). At baseline, most participants (*n* = 104; 73.2%) were classified as having a severe AUD, with the remaining participants falling into the moderate (*n* = 25; 17.6%) and mild (*n* = 13; 9.2%) categories. The proportion of participants in each severity category did not significantly differ by treatment assignment. Changes in AUD severity over time are displayed in Fig. 3, which shows the percentage of participants categorized as mild, moderate, and severe AUD at screening, 12-month follow-up, and 24-month follow-ups. Most participants (70%) achieved at least a one-level reduction in AUD severity and almost half (48%) achieved a two-level reduction in AUD severity measured at the 24-month follow-up.

#### 3.2.2 Online questionnaires

At 12-month follow-up, participants in LIII had reduced their levels of craving (PACS) and depression (MADRS-S) to a significantly greater extent compared to HIII (PACS: HIII = 10.28 versus LIII = 6.70, *p* = .006, g = 0.578; MADRS-S: HIII = 10.80 versus 7.43, *p* = .018, g = 0.494); these differences did not appear in any other measure or at any other time point, see Table 5. At the 24-month follow-up, participants in LIII had lower AUDIT scores compared to HIII (HIII = 12.61 versus LIII = 8.88, *p* = .018, g = −0.52); again, this difference did not appear at any other time point.

**Table 5:** GEE analysis of secondary outcomes measured with online questionnaires at screening, post-treatment and long-term follow-ups.

		Screening	Post	6-Month	12-Month	24-Month
Alcohol problems (AUDIT)	HIII, m (SE)	23.21 (0.60)	14.81 (0.85)	15.12 (0.89)	12.89 (1.07)	12.61 (1.32)
	LIII, m (SE)	23.89 (0.57)	16.30 (0.94)	13.67 (1.01)	10.11 (1.02)	8.88 (0.87)
	Sign.	*p* = .411	*p* = .239	*p* = .279	*p* = .059	*p* = .018
	Effect size	*g* = 0.14	*g =* 0.21	*g =* −0.21	*g =* −0.39	*g =* −0.52
Self-efficacy (AASES)	HIII, m (SE)	2.31 (0.08)	3.06 (0.10)	3.19 (0.15)	3.22 (0.15)	3.33 (0.17)
	LIII, m (SE)	2.40 (0.09)	3.16) (0.10)	3.37 (0.12)	3.56 (0.13)	3.46 (0.18)
	Sign.	*p* = .424	*p* = .495	*p* = .257	*p* = .083	*p* = .581
	Effect size,	*g* = 0.13	*g =* −0.13	*g =* −0.18	*g =* −0.36	*g =* −0.13
Craving (PACS)	HIII, m (SE)	17.18 (0.55)	10.75 (0.66)	10.65 (0.70)	10.28 (1.03)	9.10 (1.10)
	LIII, m (SE)	17.93 (0.61)	11.15 (0.78)	9.74 (0.83)	6.70 (0.78)	7.27 (1.02)
	Sign.	*p* = .364	*p* = .694	*p* = .402	*p* = .006	*p* = .223
	Effect size,	*g =* 0.15	*g =* 0.071	*g =* 0.160	*g =* 0.578	*g =* 0.286
Depression (MADRS-S)	HIII, m (SE)	17.17 (0.77)	8.67 (0.91)	10.05 (1.11)	10.80 (1.11)	10.95 (1.36)
	LIII, m (SE)	15.86 (0.86)	9.16 (0.94)	9.11 (0.99)	7.43 (0.89)	8.04 (1.20)
	Sign.	*p* = .258	*p* = .704	*p* = .526	*p* = .018	*p* = .109
	Effect size,	*g =* 0.190	*g =* 0.07	*g =* −0.12	*g =* −0.49	*g =* −0.37
Anxiety (GAD-7)	HIII, m (SE)	6.97 (0.49)	4.27 (0.47)	4.39 (0.65)	4.44 (0.68)	4.29 (0.67)
	LIII, m (SE)	6.86 (0.59)	3.95 (0.52)	3.43 (0.53)	2.46 (0.62)	3.19 (1.00)
	Sign.	*p* = .882	*p* = .649	*p* = .254	*p* = .031	*p* = .358
	Effect size,	*g =* 0.024	*g =* 0.082	*g =* 0.216	*g =* 0.449	*g =* 0.238

HIII = High-Intensity Internet Intervention; LIII = Low-Intensity Internet Intervention; AUDIT = Alcohol Use Disorders Identification Test; MADRS = Montgomery–Åsberg Depression Rating Scale; GAD-7 = Generalized Anxiety Disorder Scale; PACS = Penn Alcohol Craving Scale; AASES = Alcohol Abstinence Self Efficacy Scale; SE = standard error.

### 3.3 Receiving other treatments

A secondary analysis of treatments in the period between post-treatment and 24-month follow-up showed that about a fifth of participants in both intervention groups accessed other treatments, 22.4% (*n* = 33) in HIII and 21.3% (*n* = 30) in LIII (χ^2^ = 0.058, *p* = .81). There were no differences in proportions of other treatments accessed between the two intervention groups, neither in the 12- nor the 24-month follow-up.

## 4 Discussion

This study aimed to investigate long-term effects of a therapist-guided high-intensity internet intervention and an unguided low-intensity internet intervention among individuals with alcohol use disorder, comparing the two groups as well as exploring within-group levels of alcohol consumption in comparison to pre-treatment and post-treatment measurements, respectively.

Our results show that, while no significant differences between the two interventions were found in either of the two primary outcomes at the 12- and 24-month follow-ups, both intervention groups maintained a lower level of alcohol consumption at both long-term follow-ups, compared to the pre-treatment measurement. However, when long-term follow-ups were compared to the post-treatment measurement, results were mixed; participants in the high-intensity intervention showed a significant increase in both number of standard drinks and heavy drinking days at the 12-month and 24-month follow-ups, while in the low-intensity intervention, participants had a significant decrease in number of standard drinks and number of heavy drinking days at 12-month follow-up, a decrease that was not significant at the 24-month follow-up. Regarding secondary outcomes, significant differences between the high and low-intensity intervention were found on craving, depression and anxiety. Sometimes these differences were present at the 12-month follow-up, sometimes at 24-month follow-up, but they never recurred over time for the same questionnaire. Some caution should be exercised in interpreting these results, however, as we did not correct for multiple testing. Further, diagnostic interview outcomes at the long-term follow-ups showed considerably fewer AUD criteria in both intervention groups, indicating that, in addition to reductions in alcohol consumption, AUD severity also decreased over time. The majority of participants achieved at least a one-level reduction in AUD severity and almost half had achieved a two-level reduction in AUD severity at the 24-month follow-up.

Our results on primary outcomes are in line with previous research of internet interventions for anxiety and depression, where symptom reductions are maintained in long-term follow-ups, but with lower effect sizes over time. For anxiety and depression, long-term effect sizes have been identified at a mean Hedge's g of 1.52 (Andersson et al., 2018), while in our study, participants in the high intensity intervention showed within-group effect sizes of *g* = 0,38–0.59 and in the low intensity intervention, participants showed effect sizes of *g* = 0.75–1.04.

Our results indicate that low-intensity internet interventions may be at least as efficient as high intensity internet interventions. There are several ways to interpret these results. Therapist guidance may have functioned as a temporary motivation booster during treatment, and some participants receiving guidance may have relapsed when it ended. It could be hypothesized that participants in LIII who did not receive any guidance, may therefore have learned to use the intervention by themselves, leading to a greater sense of self efficacy. There was, however, no significant difference between high and low intensity intervention at 12- nor 24-month follow-up regarding self-efficacy on alcohol abstinence. Another noteworthy finding is that participants in the high intensity intervention reported an increase in alcohol consumption at both long-term follow-ups compared to post-treatment, while participants in the low intensity intervention had decreased their alcohol consumption at the 12-month follow-up. One plausible reason may be the Hello-Goodbye effect, where a patient sometimes may over-exaggerate their symptoms to receive therapy and at the end of treatment, minimize or underreport their symptoms to please the clinician (Salkind, 2007). This effect could explain why participants in the high intensity intervention, who received guidance, reported lower alcohol consumption at post-treatment, which at the long-term follow-ups they had bounced back to more valid levels. Another reason for the increase in alcohol consumption in the high intensity intervention group might be that the support offered via guidance may have led to greater confidence among participants that they could maintain controlled drinking, whereas those in the low intensity intervention group may have assumed that they needed to maintain drinking levels close to complete abstinence, in order to succeed in changing their behavior over the long term. Finally, the differential attrition at the 24-month follow-up could be explained by participants in the low-intensity intervention feeling lower allegiance to the study due to the lack of guidance, where those in this group who reduced *or* increased their consumption were not motivated to participate in long-term follow-up.

### 4.1 Strengths and limitations

A significant strength is that this is the first study, to our knowledge, measuring long-term results (over one year) for internet interventions for AUD. Another strength is that we conducted telephone-based diagnostic interviews both at screening and at both long-term follow-up time-points, enabling us to follow change in AUD severity over time.

The major limitation of this study is attrition, both non-differential and differential. At the 12-month follow-up, non-differential attrition was around 40%. Although this is in line with previous research on internet interventions for problematic alcohol use (e.g. Johansson et al., 2021b; Sinadinovic et al., 2014), it still hampers conclusions. Relapse often leads to drop-out, and therefore extrapolating only from those participating in the follow-ups may be severely biased. Further, at the 24-month follow-up, attrition was substantially and significantly higher in the low-intensity group leading to differential attrition (in all, only 37% in the low-intensity intervention group participated in this follow-up). A dropout analysis was performed to examine if participants who responded to long-term follow-ups had more severe problems at screening compared to participants who did not respond to long-term follow-ups, and we indeed found that those with more severe initial problems (according to AUDIT), were significantly less likely to participate in follow-ups the 24-month follow-up. Caution is therefore called for in generally interpreting results due to attrition, but especially in interpreting results from the 24-month follow-up. Aside from attrition, it is also important to consider the issue of natural recovery. For example, epidemiological surveys show that a large number of those with previous AUD seem to recover without receiving any formal treatment [Fan et al., 2019 #869].

### 4.2 Conclusions

Both high- and low-intensity internet interventions for AUD were associated with reductions in drinking and diagnostic severity at both short- and long-term time measurements, with no significant difference between the two. Both interventions may thus have potential as easily accessible alternatives to face-to-face treatment for people with AUD who, for varying reasons, are reluctant or unable to visit a clinic, but are motivated to seek treatment online and undergo an initial assessment. However, major differential and non-differential attrition hampers interpretation of the long-term follow-ups.

### 4.3 Future research

Internet interventions based on CBT are well-tested in research settings but still novel in most clinical settings. Future studies on internet interventions on AUD should be performed in clinical settings, as has been done in one recent non-inferiority study (Johansson et al., 2021a). Future studies should also continue to examine the separate effects (both short- and long-term) of factors that might be related to outcomes, such as recruitment methods, assessment methods and different levels of guidance, preferably with factorial designs (Collins, 2018).

## Clinical trial registration details

The Regional Ethics Vetting Board in Stockholm approved the trial (2015/2014–31; amendment 2016/295–32). The original trial protocol, including short- and long-term follow-ups, was also registered at ClinicalTrials.gov (NCT02645721).

## Declaration of competing interest

NE is co-founder of the mental well-being application Remente.

## Figures and Tables

**Table:** Table Appendix. Observed values of primary and secondary outcomes measured with online questionnaires at screening, post-treatment, 6-month and long-term follow-ups.

		Screening	Post	6-month	12-month	24-month
Drinks per week	HIII, m (SD)	34.23 (17.28)	9.77 (11.26)	15.79 (14.51)	13.67 (13.94)	15.76 (17.79)
	LIII, m (SD)	33.83 (16.45)	13.26 (14.67)	13.22 (14.58)	9.65 (10.93)	10.19 (11.98)
Heavy drinking days	HIII, m (SD)	4.00 (1.94)	0.94 (1.38)	1.60 (1.88)	1.78 (2.02)	1.82 (2.18)
	LIII, m (SD)	3.99 (2.12)	1.57 (1.93)	1.54 (2.10)	1.07 (1.47)	1.15 (1.579
Alcohol problems (AUDIT)	HIII, m (SD)	23.21 (5.09)	14.81 (6,76)	15.12 (6,76)	12.89 (7,35)	12.61 (8,57)
	LIII, m (SD)	23.89 (4,86)	16.30 (7,37)	13.67 (7,50)	10.11 (7,04)	8.88 (4,50)
Self-efficacy (AASE)	HIII, m (SD)	2.31 (0.66)	3.06 (0.81)	3.19 (0.86)	3.22 (1.00)	3.33 (1.08)
	LIII, m (SD)	2.40 (0.73)	3.16) (0.82)	3.37 (0.88)	3.56 (0.89)	3.46 (0.95)
Craving (PACS)	HIII, m (SD)	17.18 (4.73)	10.75 (5.31)	10.65 (5.34)	10.28 (7.05)	9.10 (7.14)
	LIII, m (SD)	17.93 (5.20)	11.15 (6.12)	9.74 (6.13)	6.70 (5.41)	7.27 (5.31)
Depression (MADRS-S)	HIII, m (SD)	17.17 (6.60)	8.67 (7.25)	10.05 (8.45)	10.80 (7.59)	10.95 (8.60)
	LIII, m (SD)	15.86 (7.29)	9.16 (7.43)	9.11 (7.30)	7.43 (6.08)	8.04 (6.38)
Anxiety (GAD-7)	HIII, m (SD)	6.97 (4.17)	4.27 (3.75)	4.39 (4.98)	4.44 (4.64)	4.29 (4.16)
	LIII, m (SD)	6.86 (4.96)	3.95 (4.09)	3.43 (3.94)	2.46 (4.24)	3.19 (5.30)

HIII = High-Intensity Internet Intervention; LIII = Low-Intensity Internet Intervention; AUDIT = Alcohol Use Disorders Identification Test; MADRS = Montgomery–Åsberg Depression Rating Scale; GAD-7 = Generalized Anxiety Disorder Scale; PACS = Penn Alcohol Craving Scale; AASES = Alcohol Abstinence Self Efficacy Scale; SE = Standard Error.
